# Loss of SOCS3 in myeloid cells prolongs survival in a syngeneic model of glioma

**DOI:** 10.18632/oncotarget.7992

**Published:** 2016-03-08

**Authors:** Braden C. McFarland, Margaret P. Marks, Amber L. Rowse, Samuel C. Fehling, Magda Gerigk, Hongwei Qin, Etty N. Benveniste

**Affiliations:** ^1^ Department of Cell, Developmental and Integrative Biology, University of Alabama at Birmingham, Birmingham, AL, USA

**Keywords:** SOCS3, glioblastoma, JAK/STAT, macrophage, GL261

## Abstract

In glioma, microglia and macrophages are the largest population of tumor-infiltrating cells, referred to as glioma associated macrophages (GAMs). Herein, we sought to determine the role of Suppressor of Cytokine Signaling 3 (SOCS3), a negative regulator of Signal Transducer and Activator of Transcription 3 (STAT3), in GAM functionality in glioma. We utilized a conditional model in which SOCS3 deletion is restricted to the myeloid cell population. We found that SOCS3-deficient bone marrow-derived macrophages display enhanced and prolonged expression of pro-inflammatory M1 cytokines when exposed to glioma tumor cell conditioned medium *in vitro*. Moreover, we found that deletion of SOCS3 in the myeloid cell population delays intracranial tumor growth and increases survival of mice bearing orthotopic glioma tumors *in vivo*. Although intracranial tumors from mice with SOCS3-deficient myeloid cells appear histologically similar to control mice, we observed that loss of SOCS3 in myeloid cells results in decreased M2 polarized macrophage infiltration in the tumors. Furthermore, loss of SOCS3 in myeloid cells results in increased CD8^+^ T-cell and decreased regulatory T-cell infiltration in the tumors. These findings demonstrate a beneficial effect of M1 polarized macrophages on suppressing glioma tumor growth, and highlight the importance of immune cells in the tumor microenvironment.

## INTRODUCTION

On a cellular level, glioblastoma (GBM) tumors are extremely heterogeneous, consisting of resident tumor cells, tumor initiating cells, infiltrating immune cells, endothelial cells and other tumor associated stromal cells, which makes developing targeted therapies a challenge [1]. In GBM, microglia and macrophages are the largest population of tumor-infiltrating cells (5–30% of tumor mass) and are actively recruited by the tumor via the secretion of chemo-attractants including monocyte chemoattractant protein (MCP-1, also known as CCL2), stromal-derived factor-1 (SDF-1) and macrophage-colony stimulating factor (M-CSF) [2]. Although microglia are the resident tissue macrophage of the brain, macrophages are also recruited to the tumor from peripheral hematopoietic stem cell compartments. While these two cell populations (microglia and macrophages) originate from different sites, they exhibit similar functions in GBM. Depending on the stimulus, macrophages can become polarized to an M1 (pro-inflammatory) or M2 (immunosuppressive) phenotype [3]. M1 macrophages are termed classically activated, or pro-inflammatory macrophages, and are activated in response to inflammatory stimuli including LPS, IFN-γ and GM-CSF. M1 macrophages secrete pro-inflammatory cytokines, including TNF-α, IL-6 and CXCL10, present antigen to immune cells and phagocytize tumor cells. M2 macrophages are termed alternatively activated, or immunosuppressive, and are activated in response to stimuli including IL-4, IL-13, and M-CSF. M2 macrophages secrete immune-suppressive cytokines such as IL-10 and TGF-β, promote T regulatory (Treg) cell differentiation and aid in tumor progression. However, these represent two extreme examples of M1/M2 polarization. Macrophages are often presented with multiple stimuli, especially in cancer, and the M1/M2 status should be viewed as more a continuum or spectrum [4]. In GBM, the polarization and resulting phenotype of GAMs is a bit more complicated. Overall, it has generally been accepted that upon arrival at the tumor site, macrophages become polarized to an anti-inflammatory M2 phenotype, and in turn aid in tumor promotion through the secretion of immunosuppressive cytokines including TGF-β1 and IL-10, as well as the pro-angiogenic and pro-invasive cytokines VEGF and MT1-MMP, respectively [2]. However, recent studies demonstrated that in several models of GBM, GAMs exhibit a mixed M1/M2 phenotype, depending on the time and stage of disease [5–9].

The Janus Kinase (JAK)/Signal Transducer and Activator of Transcription (STAT) pathway is commonly activated during inflammatory and immune responses and plays an important role in the response of myeloid cells to various stimuli [10]. Binding of a cytokine, such as IFN-γ or IL-6, activates the receptors and in turn the associated intracellular JAK kinases become tyrosine phosphorylated. The JAKs then phosphorylate STAT transcription factors, which dimerize, translocate to the nucleus and induce gene expression [11]. There are four JAK kinases (JAK1, JAK2, JAK3, and TYK2) and a total of seven STAT transcription factors (STAT 1, 2, 3, 4, 5a, 5b, and 6) [12]. In addition to pro-survival genes, JAK/STAT activation also induces the expression of Suppressor Of Cytokine Signaling (SOCS) proteins. SOCS proteins are negative regulators of the JAK/STAT pathway, and prevent prolonged, unregulated activation of the JAK/STAT pathway [3]. Upon cytokine stimulation and subsequent SOCS expression, SOCS proteins bind to activated JAKs and to cytokine receptors via their SH2 domains, thereby suppressing signaling through this pathway. Additionally, SOCS1 and SOCS3 contain a kinase-inhibitory domain (KIR), which acts as a pseudo-substrate for JAKs and prevents further kinase activity [3].

We have generated a mouse model in which SOCS3, a negative regulator of STAT1 and STAT3, is deleted in the myeloid cell population [13, 14]. Briefly, SOCS3^fl/fl^ mice were crossed with LysMCre mice to generate a conditional knockout mouse that is deficient for SOCS3 in the myeloid cell population (LysMCre-SOCS3^fl/fl^). Deletion of SOCS3 in macrophages resulted in enhanced basal and stimulus-induced STAT1 and STAT3 activation, and corresponded with increased expression of TNF-α, IL-6, iNOS, CCL2 and CXCL10 [13]. This gene expression pattern is reflective of the M1 pro-inflammatory phenotype. Interestingly, when using M2 stimuli such as IL-10 or IL-4, there was not enhanced STAT3 or STAT6 signaling, respectively, in the SOCS3-deficient macrophages [14, 15]. These data suggest that that loss of SOCS3 results in an enhanced M1 phenotype in macrophages. Additionally, *in vivo* studies confirmed polarization to the pro-inflammatory M1 macrophage phenotype. LysMCre-SOCS3^fl/fl^ mice exhibited enhanced inflammatory responses in models of Multiple Sclerosis and LPS-induced sepsis compared to SOCS3^fl/fl^ control mice [13, 14]. Overall, these studies confirm that loss of SOCS3 in myeloid lineage cells promotes a pro-inflammatory M1 phenotype, and this model could be used to determine the role of M1 macrophages in additional disease states.

For this study, we have utilized the LysMCre-SOCS3^fl/fl^ M1 model in combination with the GL261 syngeneic model of glioma. The GL261 model is widely used for immunotherapeutic studies and is the most appropriate for the studies described herein [16]. Murine GL261 cells were injected into the brains of SOCS3^fl/fl^ and LysMCre-SOCS3^fl/fl^ mice (termed SOCS3^−/−^) in order to establish an orthotopic M1 model of glioma. We found that SOCS3-deficient bone marrow-derived macrophages (BMDM) display enhanced and prolonged expression of pro-inflammatory M1 cytokines when exposed to GL261 tumor cell conditioned medium *in vitro*. Moreover, we found that deletion of SOCS3 in the myeloid cell population delays intracranial tumor growth and increases survival of mice bearing orthotopic glioma tumors *in vivo*. We observed that loss of SOCS3 in myeloid cells results in decreased M2 polarized macrophage infiltration, increased CD8^+^ T-cell and decreased Treg infiltration in the tumors. This study unveils a novel model of suppressing glioma tumor growth through manipulating GAM function, and highlights the importance of targeting GAMs in glioma tumors.

## RESULTS

### Loss of SOCS3 prolongs STAT3 activation in response to GL261 tumor cell conditioned medium

**Figure 1:**
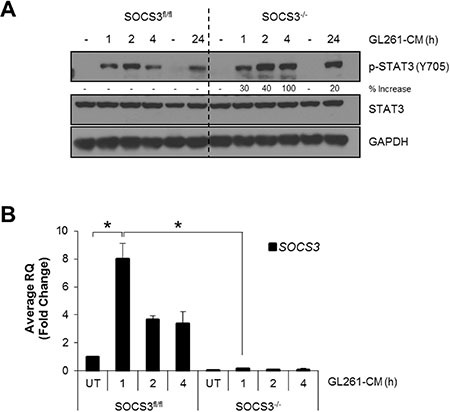
SOCS3-deficient macrophages exhibit prolonged activation of STAT3 when exposed to GL261 conditioned medium (**A** and **B**) SOCS3^fl/fl^ and SOCS3^−/−^ BMDM were harvested from the femurs of 7–8 week old mice and cultured in RPMI 1640 containing 10% FBS and 10 ng/ml murine M-CSF for 5–7 days to expand. Cells were plated and at 24 h treated with GCM (50% volume) for the indicated times. Cells were lysed and immunoblotted with the indicated Abs (A) or RNA was isolated, cDNA generated and qRT-PCR performed for the indicated genes (B) Densitometric analysis is displayed as percent increase of SOCS3^−/−^ compared to control SOCS3^fl/fl^ macrophages. For example, at 1 h the SOCS3^−/−^ macrophages display 30% higher p-STAT3 than the corresponding SOCS3^fl/fl^ 1 h time point. *p < 0.05. Data are shown as mean ± S.D.

We have previously shown that macrophages deficient in SOCS3 display prolonged STAT1/STAT3 activation and increased M1 gene expression in response to LPS, IL-6 and IFN-γ stimulation [14]. Therefore, we sought to determine the response of SOCS3^fl/fl^ and SOCS3^−/−^ macrophages when using GL261 conditioned medium (GCM) as the stimulus. To generate GCM, GL261 glioma cells were plated in serum free DMEM/F12 media for 24 h and supernatant collected. BMDM were isolated from SOCS3^fl/fl^ and SOCS3^−/−^ mice and treated with GCM (50% volume) for various times. As shown in Figure 1A, both SOCS3^fl/fl^ and SOCS3^−/−^macrophages displayed STAT3 phosphorylation in response to GCM. However, SOCS3^−/−^ macrophages displayed an enhanced activation at 4 h, which remained elevated at 24 h. This indicates that loss of SOCS3 prolongs STAT3 activation in response to GCM. STAT1 phosphorylation was enhanced in SOCS3^−/−^ macrophages when exposed to GCM; however, total levels of STAT1 protein were also enhanced, and densitometric quantification reveals no elevated STAT1 activation in SOCS^−/−^ macrophages compared to SOCS3^fl/fl^ cells (Supplementary Figure 1). Treatment of SOCS3^fl/fl^ macrophages with GCM induced mRNA expression of *SOCS3* (Figure 1B). As expected, SOCS3^−/−^ macrophages do not constitutively or inducibly express *SOCS3*, confirming the conditional deletion. Thus, treatment of SOCS3^−/−^ macrophages with GCM promotes enhanced and prolonged STAT3 activation, due to the absence of SOCS3.

### GL261 cells secrete both M1 and M2 polarizing cytokines

GBM cells secrete numerous cytokines, most of which are immunosuppressive and maintain the growth of the tumor [2]. We tested the levels of both M1 and M2 polarizing cytokines secreted by GL261 cells. Cells were plated in serum free DMEM/F12 medium for 24 h, and supernatants were collected and analyzed by Multiplex ELISA. We found that GL261 cells secrete M1 (GM-CSF, IL-6, IFN-γ) and M2 (GM-CSF, IL-13, M-CSF, IL-10 and IL-4) polarizing cytokines *in vitro*, as well as numerous other cytokines and chemokines (Table 1). Several STAT family members are activated in response to these cytokines and are shown in Table 1. These data indicate that GL261 glioma cells secrete numerous cytokines that can polarize macrophages to the M1 and/or M2 phenotype and have the potential to activate various STAT transcription factors.

**Table 1:** Multiplex ELISA of GL261 supernatants

Analyte	pg/ml	Polarization	STAT Activation
LIX	2729.56		
IL-1α	344.90		
IL-13	223.44	M2	STAT6
IP-10	214.01		
Eotaxin	209.16		
G-CSF	172.90		
IL-9	154.57		
GM-CSF	76.98	M1/M2	STAT5
MIP-2	68.66		
IL-15	64.96		
MIG	55.63		
KC	42.37		
MCP-1	34.60		
MIP-1α	34.22		
IL-6	24.65	M1	STAT3
IL-1b	24.07		
MIP-1β	21.75		
M-CSF	19.41	M2	
RANTES	16.80		
IL-12 (p70)	15.52		STAT4
IL-12 (p40)	14.63		STAT4
IL-10	10.11	M2	STAT3
IL-7	7.68		
IL-2	7.36		STAT5
TNF-α	6.80		
IFN-γ	6.35	M1	STAT1
IL-5	5.64		
IL-17	4.72		
LIF	2.98		STAT3
IL-3	2.94		
VEGF	2.38		
IL-4	1.84	M2	STAT6

### SOCS3^−/−^ macrophages display an enhanced M1 response when exposed to GCM

**Figure 2:**
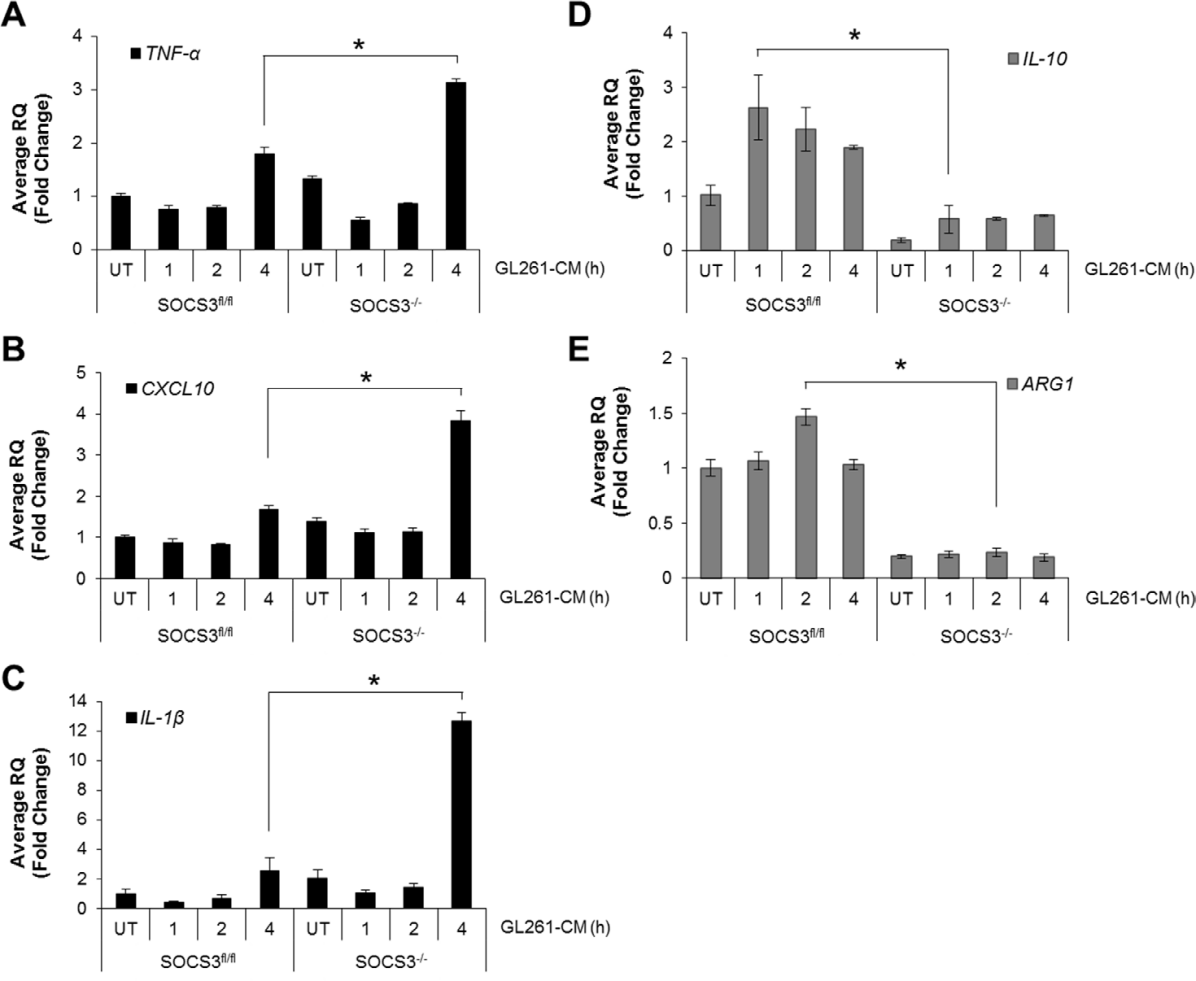
SOCS3^−/−^ macrophages display enhanced M1 gene expression when exposed to GL261 conditioned medium (**A**–**E**) SOCS3^fl/fl^ and SOCS3^−/−^ BMDM were harvested from the femurs of 7–8 week old mice and cultured in RPMI 1640 containing 10% FBS and 10 ng/ml murine M-CSF for 5–7 days to expand. Cells were plated and at 24 h treated with GL261 conditioned medium (50% volume) for the indicated times. RNA was isolated, cDNA generated and qRT-PCR performed for the indicated genes. *p < 0.05. Data are shown as mean ± S.D.

M1 macrophages are characterized by an increase in pro-inflammatory gene expression [3]. BMDM from SOCS3^fl/fl^ and SOCS3^−/−^ mice were treated with GCM for 4 h, and mRNA expression levels of M1 and M2 markers measured. Following treatment with GCM, SOCS3^fl/fl^ macrophages expressed mRNA for *TNF*- α, *CXCL10* and *IL-1β* (M1 genes) by 4 h, whereas SOCS3^−/−^ macrophages displayed significantly higher expression levels at 4 h (Figure 2A–2C). In addition, SOCS3^fl/fl^ macrophages expressed *IL-10* and *Arginase-1* (M2 genes) in response to treatment with GCM, whereas SOCS3^−/−^ macrophages displayed significantly lower levels (Figure 2D and 2E). Of note, the SOCS3^−/−^ macrophage basal (untreated) levels of the M2 genes were lower than that of SOCS3^fl/fl^ macrophages. These findings indicate that in response to secreted tumor cytokines, macrophages that lack SOCS3 have an increased M1 response.

### Loss of myeloid SOCS3 prolongs *in vivo* survival

**Figure 3:**
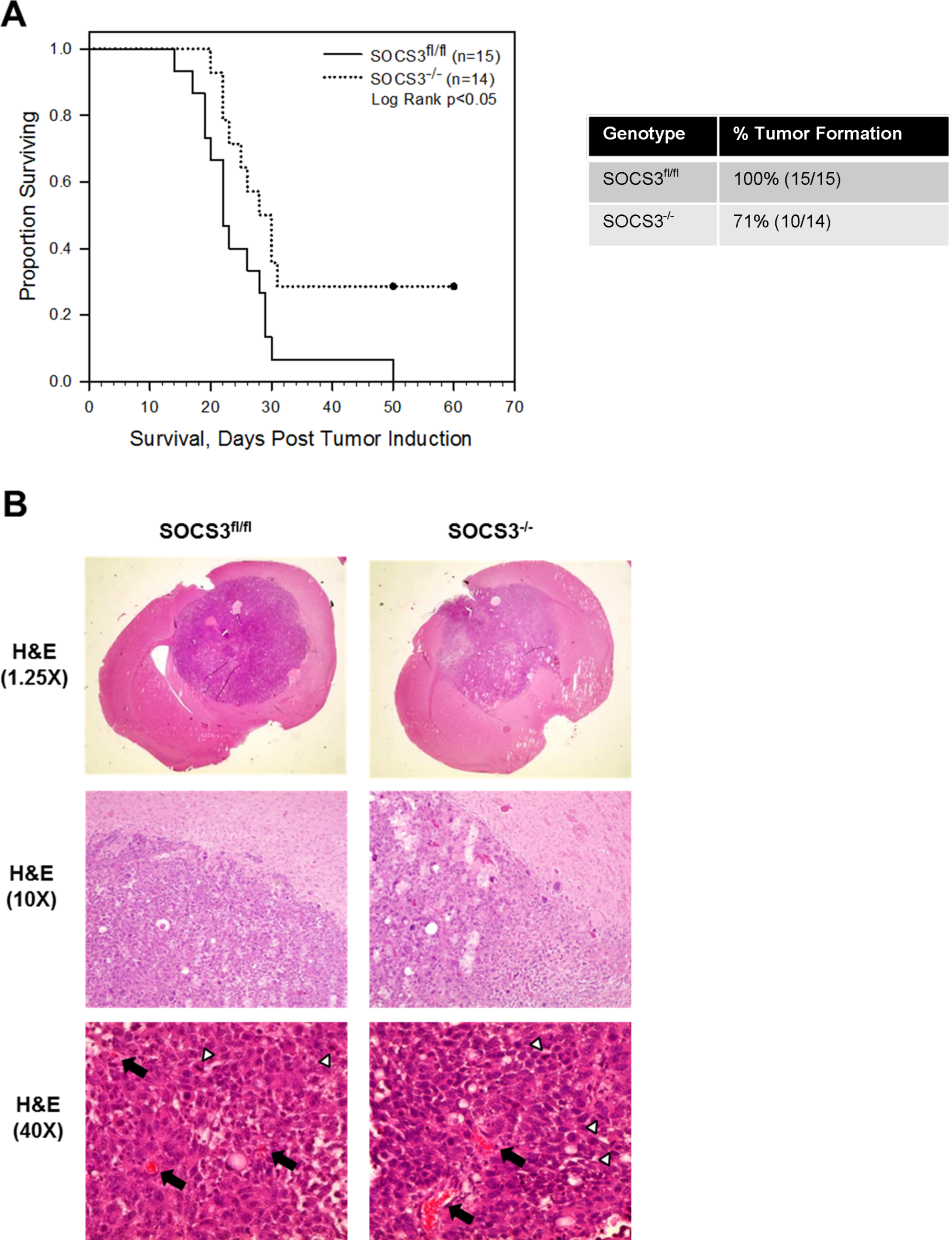
Deletion of SOCS3 in myeloid cells prolongs *in vivo* survival (**A**) SOCS3^fl/fl^ and SOCS3^−/−^ mice were injected intracranially with GL261 cells (1 × 10^6^ cells/5 μl). Mice were monitored for survival and euthanized at moribund. Combination of two independent experiments are shown. Log Rank *p* < 0.05. (**B**) At death, (times varied as shown in survival curve) brains from SOCS3^fl/fl^ and SOCS3^−/−^ mice injected with GL261 cells were formalin fixed, paraffin embedded, sectioned and H & E stained. Representative images are shown from each group. Black arrows indicate blood vessels and white arrow heads indicate mitotic figures.

Our data thus far indicate that loss of SOCS3 in macrophages results in an enhanced M1, or pro-inflammatory, anti-tumor phenotype when exposed to GCM. Therefore, we tested the ability of SOCS3^−/−^ macrophages to regulate tumor growth *in vivo* in an intracranial model of glioma. GL261 cells were injected into the brains of SOCS3^fl/fl^ and SOCS3^−/−^ mice. Mice were monitored for physical signs of tumor burden and were euthanized at moribund and the brains removed for histology. SOCS3^−/−^ mice exhibited a significantly prolonged survival compared to SOCS3^fl/fl^ mice (Figure 3A). SOCS3^−/−^ mice also exhibited decreased tumor formation (71%; 10/14) when compared to SOCS3^fl/fl^ mice (100%; 15/15) (Figure 3A). The intracranial tumors from SOCS3^fl/fl^ and SOCS3^−/−^ mice appear histologically similar in size and morphology (Figure 3B; 1.25× and 10×), and the numbers of mitotic figures and blood vessel density were quantified (Figure 3B; 40× and Supplementary Figure 2). Interestingly, at the time of death, tumors from the SOCS3^−/−^ mice displayed significantly increased mitotic figures and microvessel (MV) density compared to tumors from the SOCS3^fl/fl^ mice, possibly due to overcoming resistance and escaping an immune response in the SOCS3^−/−^ mice (Supplementary Figure 2).

The survival data suggest tumor growth is inhibited in mice with SOCS3 deletion in myeloid cells. Therefore, we evaluated the growth of intracranial tumors in real time by bioluminescent imaging (BLI). GL261 cells were labeled with firefly luciferase (GL261-Luc) and injected into the brains of SOCS3^fl/fl^ and SOCS3^−/−^ mice. On days 5–9 post tumor injection, tumor volume was similar between SOCS3^fl/fl^ and SOCS3^−/−^ mice (Supplementary Figure 3A and 3B). On days 13–16 post tumor injection, SOCS3^−/−^ mice display decreased tumor volume, although not statistically significant, when compared to SOCS3^fl/fl^ tumor volume (Supplementary Figure 3C and 3D). This suggests that the prolonged survival observed in the SOCS3^−/−^ mice is not exclusively due to inhibition of tumor volume, but alternative mechanisms including altered immune cell function within the tumors may be responsible for the prolonged survival.

### Numbers of infiltrating myeloid cells are similar between SOCS3^fl/fl^ and SOCS3^−/−^ tumor bearing mice

**Figure 4:**
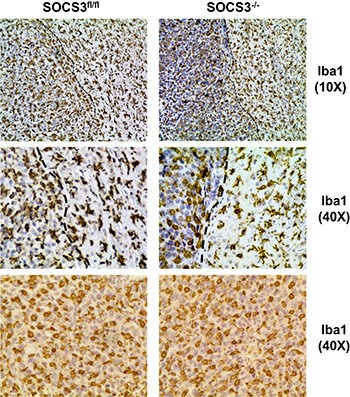
GAM infiltration and morphological activation is similar between SOCS3^fl/fl^ and SOCS3^−/−^ tumor bearing mice SOCS3^fl/fl^ and SOCS3^−/−^ mice were injected intracranially with GL261 cells (1 × 10^6^ cells/5 μl). Mice were monitored for survival and euthanized at moribund. Brains were formalin fixed, paraffin embedded, sectioned and stained with Iba1. Representative images are shown from each group. Top and middle panels, 10× and 40× Iba1 staining of brains with both tumor and normal brain shown. Black dotted line denotes border between tumor (left) and normal brain (right). Bottom panels, 40× Iba1 staining of intracranial tumor section.

Myeloid cells, which include monocytes, neutrophils and macrophages, along with resident microglia, are the largest population of infiltrating cells in GBM tumors [2]. Therefore, we sought to determine if the levels of infiltrating myeloid cells varied in intracranial tumors in SOCS3^fl/fl^ and SOCS3^−/−^ mice. GL261 cells were injected into the brains of SOCS3^fl/fl^ and SOCS3^−/−^ mice. At death, brain sections were stained with IbaI to label infiltrating GAMs in the brains and tumors of the mice. Iba1 stains microglia in the normal brain as well as GAMs in the tumor [6]. Both SOCS3^fl/fl^ and SOCS3^−/−^ mice have a robust infiltration of GAMs in the tumors (Figure 4). Morphologically, both SOCS3^fl/fl^ and SOCS3^−/−^ brain sections contain GAMs that appear amoeboid, or activated, in the tumor (left of the black dotted line) when compared to the ramified or resting microglia seen in the normal brain (right of the dotted line) (Figure 4, top and middle panels), which is comparable to what others have reported [17].

**Figure 5:**
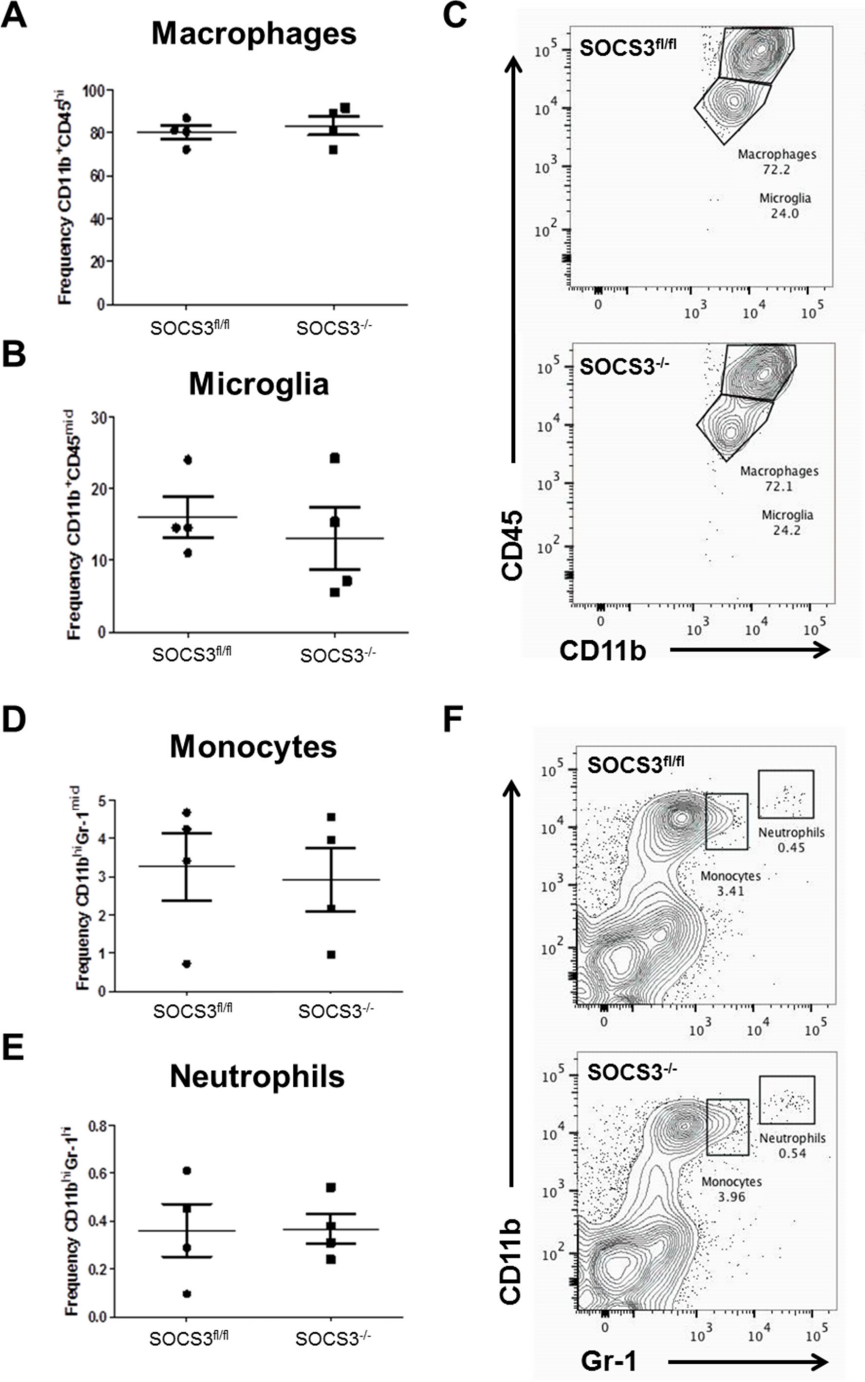
Loss of SOCS3 in myeloid cells does not affect the quantity of myeloid cell tumor infiltration (**A–F**) SOCS3^fl/fl^ (*n* = 4) and SOCS3^−/−^ (*n* = 4) mice were injected intracranially with GL261 cells (1 × 10^6^ cells/5 μl). Mice were euthanized on day 15, and mononuclear cells were isolated from the brains/tumors and analyzed by flow cytometry for macrophages (CD11b^+^ F4/80^+^ CD45^hi^), microglia (CD11b^+^ F4/80^+^ CD45^mid^), monocytes (CD11b^mid^ Gr-1^mid^), or neutrophils (CD11b^hi^ Gr-1^hi^), and were gated and quantified. Data are shown as mean ± S.D.

To more appropriately quantify the numbers of myeloid cells in tumors of the mice, flow cytometry was employed. GL261-Luc cells were injected into the brains of SOCS3^fl/fl^ and SOCS3^−/−^ mice. At day 15, all mice were euthanized to determine the levels of infiltrating GAMs in the brains/tumors of the mice. Mononuclear cells were isolated and stained with Abs to CD11b, Gr-1, F4/80 and CD45 and the percentage of macrophages (CD11b^+^ F4/80^+^ CD45^hi^), microglia (CD11b^+^ F4/80^+^ CD45^mid^), monocytes (CD11b^mid^ Gr-1^mid^), or neutrophils (CD11b^hi^ Gr-1^hi^), and were gated and quantified as previously described [18, 19]. As shown in Figure 5, there was not a statistically significant difference in the number of infiltrating macrophages, microglia, monocytes or neutrophils between tumors from SOCS3^fl/fl^ and SOCS3^−/−^ mice.

### Decreased infiltration of Arg1^+^ GAMs in intracranial tumors of SOCS3^−/−^ mice

**Figure 6:**
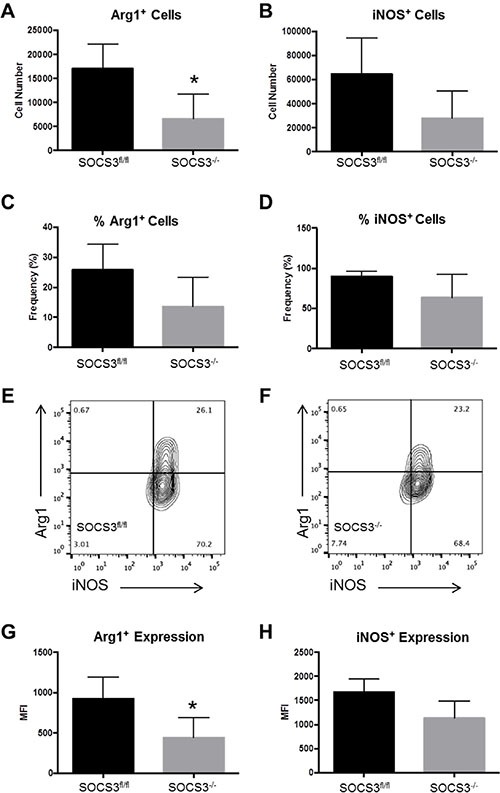
SOCS3^−/−^ mice with intracranial tumors have diminished numbers of infiltrating Arg1^+^ M2 polarized cells compared to SOCS3^fl/fl^ mice SOCS3^fl/fl^ mice (*n* = 4) and SOCS3^−/−^ mice (*n* = 4) were injected with GL261-Luc cells (1 × 10^6^ cells/5 μl). Mice were euthanized on day 14, and mononuclear cells were isolated from the brains/tumors and analyzed by flow cytometry for Arg1 expression (CD45^+^ CD11b^+^ Arg1^+^) and iNOS expression (CD45^+^ CD11b^+^ iNOS^+^). *p < 0.05. Data are shown as mean ± S.D.

Given that the absolute numbers of GAMs did not differ between SOCS3^fl/fl^ and SOCS3^−/−^ mice, we speculated there may be functional differences in the GAM population that are responsible for the prolonged survival. Therefore, we tested M1/M2 markers in GAMs in the *in vivo* intracranial model. GL261-Luc cells were injected into the brains of SOCS3^fl/fl^ and SOCS3^−/−^ mice. At day 14, all mice were euthanized to determine the levels of M1/M2 GAMs in the brains/tumors of the mice. Mononuclear cells were isolated and stained with Abs to CD45, CD11b, Arg1, and iNOS and the percentage of M2 GAMs (CD45^+^ CD11b^+^ Arg1^+^) and M1 GAMs (CD45^+^ CD11b^+^ iNOS^+^) were gated and quantified. We found that tumors in the SOCS3^−/−^ mice contained significantly decreased numbers of Arg1^+^ (M2) GAMs compared to SOCS3^fl/fl^ mice, but no difference in the number of iNOS^+^ (M1) GAMs (Figure 6A and 6B). However, there were no significant differences between the frequency of Arg1^+^ or iNOS^+^ GAMs in the tumors of SOCS3^fl/fl^ compared to SOCS3^−/−^ mice (Figure 6C and 6D). Interestingly, we found that the Arg1^+^ cells in both SOCS3^fl/fl^ and SOCS3^−/−^ tumors were also positive for iNOS, indicating that the M1/M2 status of the GAMs in our model is not mutually exclusive, and a mixed phenotype is observed (Figure 6E and 6F, upper right quandrant). Lastly, the expression level (MFI) of Arg1 in GAMs was significantly decreased in the tumors of the SOCS3^−/−^ mice compared to SOCS3^fl/fl^ mice (Figure 6G), but iNOS expression in GAMs did not reach statistical significance (Figure 6H). Thus, *in vivo* SOCS3 deletion in myeloid cells results in decreased numbers of Arg1^+^ GAM infiltration as well as overall decreased Arg1^+^ expression in the tumors of SOCS3^−/−^ mice compared to SOCS3^fl/fl^ mice.

### Increased infiltration of CD8^+^ T-cells and reduced infiltration of Tregs in intracranial tumors of SOCS3^−/−^ mice

**Figure 7:**
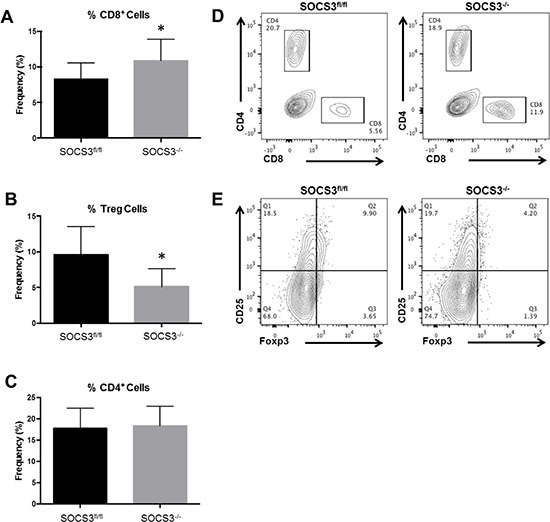
Loss of SOCS3 in myeloid cells results in increased CD8^+^ T-cell and decreased Treg infiltration in tumors (**A** and **C**) SOCS3^fl/fl^ (*n* = 11) and SOCS3^−/−^ (*n* = 10) mice were injected intracranially with GL261-Luc cells (1 × 10^6^ cells/5 μl). Mice were euthanized on days 13–14 for analysis of infiltrating CD8^+^ T-cells (CD8^+^) and CD4^+^ T-cells (CD4^+^) in the brains and quantified by flow cytometry. Combination of two experiments are shown. *p < 0.05. (**B**) SOCS3^fl/fl^ (*n* = 9) and SOCS3^−/−^ (*n* = 9) mice were injected intracranially with GL261-Luc cells (1 × 10^6^ cells/5 μl). Mice were euthanized on days 13 or 21 for analysis of infiltrating Tregs (CD4^+^CD25^+^Foxp3^+^). Combination of two experiments are shown. *p < 0.05. Data are shown as mean ± S.D. (**D** and **E**) Representative histograms of CD4^+^, CD8^+^ and Treg populations are shown for each genotype.

The presence of immunosuppressive Tregs is abundant in GBM tumors and corresponds to an inhibition of cytotoxic CD8^+^ T-cell function [20]. As GAMs are known to suppress anti-tumor T-cell mechanisms [4], we determined the levels of T-cell subsets in our model. GL261-Luc cells were injected into the brains of SOCS3^fl/fl^ and SOCS3^−/−^ mice. On days 13–21, to ensure similar tumor volumes as measured by BLI between experiments, mice were euthanized and numbers of infiltrating T-cells analyzed by flow cytometry. We found that SOCS3^−/−^ tumor bearing mice have significantly increased CD8^+^ T-cell infiltration and significantly decreased Treg infiltration in the brains/tumors (Figure 7A–7B, 7D–7E). There were no differences in the percentage of CD4^+^ T-cells in the mice (Figure 7C and 7D). This observation indicates that in addition to reduced numbers of M2 GAMs in SOCS3^−/−^ mice, an increased cytotoxic T-cell response may aid in overall survival.

## DISCUSSION

In glioma, it is widely accepted that the most common infiltrating cells in the tumor are macrophages and microglia [21–23]. However, methods to label and distinguish microglia from peripheral macrophages (GAMs) are not as concise [21]. The most commonly used method to distinguish resident microglia versus infiltrating GAMs from the periphery is the expression of CD45, with microglia displaying mid to low CD45 expression and GAMs displaying high CD45 expression [23]. Using this method, we observed a much higher frequency of GAMs infiltrating the brains/tumors of both SOCS3^fl/fl^ and SOCS3^−/−^ mice compared to resident microglia (Figure 5A–5C). Others have reported similar infiltration of peripheral GAMs in glioma tumors [24, 25]. In order to evaluate the role of SOCS3/STAT3 in myeloid cells in our model, we employed the LysMCre conditional mouse model. Recently, reports have indicated that the LysMCre efficiency in microglia is less than 25% [26–28]; thus, in our model, some expression of SOCS3 would be retained in microglia following the conditional deletion and theoretically still aide tumor growth. Regardless, we observed that loss of SOCS3 in the myeloid population produced a phenotype that significantly prolonged survival of mice bearing intracranial tumors (Figure 3A). The SOCS3^−/−^ mice also exhibited reduced tumor incidence (Figure 3A), indicating that the loss of SOCS3 in the myeloid cell population may aide in tumor rejection. However, the observation that there was no statistically significant difference in tumor volume could be explained by the role of microglia, as it has been shown that microglia aide in tumor invasion [29], which would be separate from the anti-tumor effects of the SOCS3 deficient GAMs in our model. Overall, the anti-tumor effects leading to prolonged survival in the SOCS3^−/−^ mice we observed can be attributed to GAMs, which have infiltrated from the periphery and are the major population of infiltrating cells.

To date, the role of JAK/STAT signaling in GBM macrophages remains relatively uncharacterized. For example, it has been postulated that STAT1 activation is associated with M1 polarization and STAT3 activation is associated with M2 polarization [23, 30]. In addition, Zhang et al. showed that intratumoral injection of STAT3 targeted siRNA promoted M1 activation [31]. Although there is evidence that STAT3 is activated in GAMs and may be responsible for the M2 phenotype, our data demonstrate that STAT3 also influences an M1 anti-tumor response. We found that BMDMs deficient for SOCS3 exhibited an enhanced STAT3 activation that led to increased expression of M1 proinflammatory cytokines *in vitro*. Furthermore, our *in vivo* data suggest that enhanced GAM STAT3 activation leads to decreased M2 GAM infiltration in tumors of SOCS3^fl/fl^ mice compared to SOCS3^−/−^ mice *in vivo*. Thus, the exact role and mechanisms of how macrophages can regulate GBM and how the JAK/STAT or other signaling pathways are utilized by these macrophages remains controversial.

Pro-inflammatory M1 macrophages are becoming an exciting therapeutic target in cancer because of their anti-tumor functional properties. Moreover, in patients with GBM, Zeiner et al., found that an M1 polarized immune milieu is associated with prolonged survival [32]. Thus, discovering ways to promote M1 macrophage polarization in tumors is being examined as a relevant therapeutic avenue. Recent reports indicate that amphotericin administration or CSF-1R inhibition promotes M1 activity in GBM [33, 34]. Additionally, Lisi et al., found that mTOR kinase inhibitors promoted glioma activated microglia to express an M1 phenotype [35]. In a rat model of glioma, dopamine treatment inhibited tumor growth through reprogramming of M2-polarized macrophages to an M1 phenotype [36]. In other cancer models, various agents including low dose irradiation and phosphatidylserine antibody treatment resulted in promoting a pro-inflammatory M1 phenotype in GAMs that decreased tumor growth [37, 38]. Thus, it is becoming increasingly obvious that promoting anti-tumor macrophages is beneficial, but the mechanisms of how to maintain the M1 anti-tumor phenotype *in vivo* remains largely unknown.

The terminology and characterization of M1/M2 macrophages is also becoming increasingly complicated in the context of cancer. Recent studies demonstrated that in several models of cancer including GBM, GAMs exhibit a mixed M1/M2 phenotype, depending on the time and stage of disease. For example, when freshly isolated GAMs from human GBM patients were compared to M1 and M2 polarized human macrophages, it was observed that GAMs from GBM patients expressed both M1 and M2 markers simultaneously [6]. More recently, Szulzewsky et al., performed an extensive microarray analysis of GAMs from two murine models of glioma and compared to existing M1/M2 databases [8]. They observed that GAMs from both murine glioma models display a gene expression pattern that only partly overlapped with specific M1 and M2 subsets, and that the GAM expression profile can be considered a unique glioma-associated phenotype, independent of traditional macrophage subsets. When evaluating the evolution of GAM function during the course of disease, it was reported that GAMs display a M2 phenotype early, but switch to a mixed M1/M2 phenotype later in a rat model of glioma [7]. Furthermore, it has been demonstrated that in the GL261 model of glioma, GAMs are closely similar to myeloid-derived suppressor cells (MDSC) in function and also exhibit a mixed M1/M2 phenotype [5]. The role of MDSCs, an immunosuppressive myeloid progenitor cell population [39], is not detailed in this model. Because the majority of the myeloid cells infiltrating the brains/tumors of mice were Gr-1^−^ (Figure 5D–5F), and MDSCs are largely Gr-1^+^, we did not examine their role.

In our model, we found that the overall total number of Arg1^+^ GAMs were significantly decreased in tumors of SOCS3^−/−^ mice compared to SOCS3^fl/fl^ mice. Interestingly, we observed that Arg1^+^ GAMs were almost exclusively iNOS^+^ as well in tumors of both SOCS3^fl/fl^ and SOCS3^−/−^ mice. This indicates that similar to what others have reported, GAMs in our model display a mixed M1/M2 phenotype. On the other hand, we observed that the overall expression, i.e., amount of Arg1 per cell, was decreased in tumors of SOCS3^−/−^ compared to SOCS3^fl/fl^ mice. Thus, deletion of SOCS3 in myeloid cells resulted in decreased total numbers of Arg1^+^ GAMs as well as decreased overall expression in GAMs. The exact role of arginase expression in GAM function has yet to be fully appreciated and will be examined in the future.

Glioma tumors express tumor-associated antigens that should be detectable to immune clearance [40, 41]. However, due to the immunosuppressive environment, these tumors are allowed to continue growth and proliferation. In cancer, it is widely accepted that the predominance of Tregs in the tumor help aid in immunosuppression [41]. Expression of forkhead transcription factor 3 (Foxp3) and suppression of T-cell mediated immunity are exclusive characteristics of Tregs [42]. In GBM, Tregs suppress CD4^+^ and CD8^+^ T-cell activation and are considered a major contributor to tumor progression [41]. In this study, we found that SOCS3^−/−^ tumors exhibited decreased Treg infiltrates as well as a reciprocal increase in CD8^+^ T-cell infiltrates. This is an exciting observation, and indicates that therapeutic manipulation of GAMs can enhance T-cell mediated anti-tumor immune responses in GBM.

In conclusion, the use of targeted small molecule inhibitors in GBM has been unsuccessful in the clinical setting, and alternative methods of combating tumor growth are being considered [1]. This report describes a novel mouse glioma model where the infiltrating GAMs promote an anti-tumor response. This model is a genetic model (conditional deletion of SOCS3 in myeloid cells) and therefore does not rely on small molecule inhibitors or other treatments that could yield toxicities or off-target effects to induce the therapeutic response. This allows for a definitive method of understanding the role of macrophages in GBM, as well as future experiments determining the therapeutic potential of GAMs when combined with additional anti-cancer therapies.

## MATERIALS AND METHODS

### Ethics statement

Investigation has been conducted in accordance with the ethical standards and according to the Declaration of Helsinki and according to national and international guidelines and has been approved by the authors' institutional review board.

### Cells and reagents

Murine glioma cells (GL261 and GL261-Luc) were a generous gift from Dr. G. Yancey Gillespie (UAB Brain Tumor Model Core Facility) and were grown in DMEM/F12 10% FBS, L-glutamine, and Pen/Strep as described [43]. Bone marrow derived macrophages (BMDM) were isolated and cultured as previously described [14]. For immunoblotting experiments, antibodies for phospho-STAT3 (Y705) and total STAT3 were purchased from Cell Signaling (9131; 79D7), and GAPDH from AbCam (ab8245). For IHC, rabbit anti-Iba1 was purchased from Wako (019-19741), rabbit anti-vWf from Millipore (AB7356) and rabbit anti-Ki67 from Abcam (AB15580). For flow cytometry experiments, antibodies for CD45 (30-F11), CD11b (M1/70), Gr-1 (RB6-8C5), F4/80 (BM8), CD4 (GK1.5), CD8 (53-6.7), Foxp3 (MF-14), CD25 (PC61) were purchased from BioLegend, iNOS (CXNFT) from eBioscience, and Arg1 (IC5868A) from R & D Systems [19, 44]. The mouse cytokine/chemokine multiplex ELISA assay was purchased from EMD Millipore (MPXMCyto-70K).

### Generation of GL261 conditioned medium

GL261 cells (80% confluent) were grown in DMEM/F12 media without serum for 24 h. Supernatants were collected for use in BMDM and Multiplex ELISA experiments.

### Immunoblotting

BMDMs were plated in 6-well plates and treated with GCM for the indicated times. Protein levels were quantified and equal amounts of protein were loaded on SDS-PAGE acrylamide gels and immunoblotted as described [45]. Densitometry was performed and the levels of phosphorylated STAT3 normalized to total STAT3 levels. Analyses are presented as % increase over corresponding hourly time points between the SOCS3^fl/fl^ and SOCS3^−/−^ BMDMs.

### qRT-PCR

BMDMs were plated in 6 well plates and treated with GCM for the indicated times. RNA was isolated using TriZol and cDNA generated as described [45]. cDNA was run on the Applied Biosystems platform to obtain quantitative CT values. Data are presented as Average RQ (Fold Increase) for each gene normalized to the housekeeping gene *HPRT*. The following Taqman probe/primer sets were purchased from Applied Biosystems: *SOCS3* (Mm01249143_g1), *TNF-a* (Mm00443258_m1), *CXCL10* (Mm99999072_m1), *IL-1β* (Mm01336189_m1), *IL-10* (Mm00439614_m1), *ARG1* (Mm00475988_m1), and *HPRT* (Mm00446968_m1).

### Multiplex ELISA

GL261 cells were grown in DMEM/F12 media without serum for 24 h. Supernatants were collected and run on the multiplex ELISA assay according to the manufacturer's protocol and as described [46].

### Mice

All experiments with mice were approved by the Institutional Animal Care and Use Committee of the University of Alabama at Birmingham. SOCS3 conditional knockout (SOCS3^−/−^) mice were generated by breeding SOCS3^fl/fl^ mice with mice expressing Cre recombinase under the control of the LysM promoter, in which the conditional SOCS3 allele is therefore excised in myeloid cells [13].

### Intracranial injections

Six- to 8-week-old mice were used for intracranial experiments. Mice were injected with GL261 or GL261-Luc cells as previously described [43, 47]. For survival studies, mice were monitored for signs of tumor burden and euthanized at moribund. Survival times were recorded and brains/tumors removed for analyses. For flow cytometry experiments, mice were euthanized on the indicated days and brains/tumors removed for analyses.

### Immunohistochemical staining

Mice were injected with GL261 cells and at moribund, mice were euthanized and brains removed, formalin fixed, and paraffin embedded. Sections (8 μm) were stained with hematoxylin and eosin as described [18]. For IbaI and vWf staining, sections (8 μm) were deparaffinized followed by antigen retrieval, and incubated with primary antibodies overnight at 4°C, Next, sections were incubated with SuperPicture secondary antibody for 1 h, staining detected with DAB, and nuclei counterstained with hematoxylin. Images were obtained using the EVOS XL Core Cell Imaging System, utilizing 1.25×, 10×, and 40× objective lenses in addition to the 10× eyepiece objective lens.

### Bioluminescent imaging

For the indicated experiments, mice were intracranially injected with GL261-Luc cells and tumor volume obtained using the Xenogen IVIS-100 Luminescent Imager. Mice were injected (i.p.) with luciferin and anesthetized for imaging. Region of interest (ROI) values were obtained using Living Image Software (Perkin Elmer) and plotted as average total photon counts per group. Representative images of mice are shown for each group and time point.

### Flow cytometry

Mice were intracranially injected with GL261-Luc cells, and tumor growth monitored by BLI. On the indicated days, mice were anesthetized, perfused with PBS and brains, including the tumor, were removed, collagenase digested and passed through a 100 μm cell strainer. Mononuclear cells were then obtained by a percoll gradient. Specifically, macrophages (CD11b^+^ F4/80^+^ CD45^hi^), microglia (CD11b^+^ F4/80^+^ CD45^mid^), monocytes (CD11b^mid^ Gr-1^mid^), and neutrophils (CD11b^hi^ Gr-1^hi^) were gated and analyzed for myeloid populations. For T-cell populations, CD8^+^ T-cells (CD8^+^), Tregs (CD4^+^ CD25^+^ Foxp3^+^) and CD4^+^ T-cells (CD4^+^) were gated and analyzed. For Arg1 and iNOS populations, GAMs were gated and analyzed for (CD45^+^ CD11b^+^ Arg1^+^) or (CD45^+^ CD11b^+^ iNOS^+^). Samples were run on the LSRII FACS Caliber and data analyzed by FlowJo software and displayed as total cell numbers, frequency of the population (%), or mean fluorescence intensity (MFI) as previously described [13].

### Statistical analyses

Sigma-Plot statistical analysis software was used for the statistical analyses of this manuscript. Specifically, statistical significance (*p* < 0.05) was determined using Student *t*-test for comparison of 2 values, ANOVA analysis on appropriate multivariable analyses and the Log Rank test for Kaplan–Meier survival curves.

## SUPPLEMENTARY MATERIALS FIGURES


