# Supplementary material for: Loss of SOCS3 in myeloid cells prolongs survival in a syngeneic model of glioma
Source: Oncotarget. 2016 Mar 8;7(15):20621–35. doi: 10.18632/oncotarget.7992 (PMC4991480; doi:10.18632/oncotarget.7992)
Supplement: Supplementary file 1 [file oncotarget-07-20621-s001.pdf]

Loss of SOCS3 in myeloid cells prolongs survival in a syngeneic model of glioma

Supplementary Materials

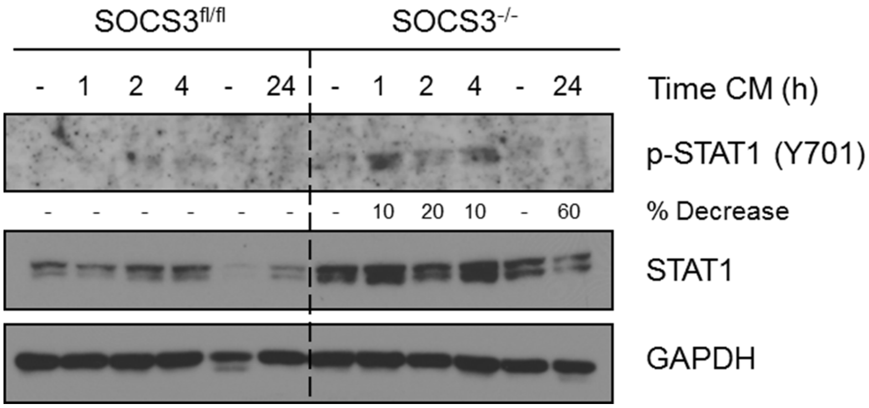

Supplementary Figure S1: SOCS3-deficient macrophages do not exhibit activation of STAT1 when exposed to GL261 conditioned medium. $SOCS3^{fl/fl}$ and $SOCS3^{-/-}$ BMDM were harvested from the femurs of 7–8 week old mice and cultured in RPMI 1640 containing 10% FBS and 10 ng/ml murine M-CSF for 5–7 days to expand. Cells were plated and at 24 h treated with GCM (50% volume) for the indicated times. Cells were lysed and immunoblotted with the indicated Abs. Densitometric analysis is displayed as percent decrease of $SOCS3^{-/-}$ compared to control $SOCS3^{fl/fl}$ macrophages. For example, at 1 h the $SOCS3^{-/-}$ macrophages display 10% less p-STAT1 than the corresponding $SOCS3^{fl/fl}$ 1 h time point.

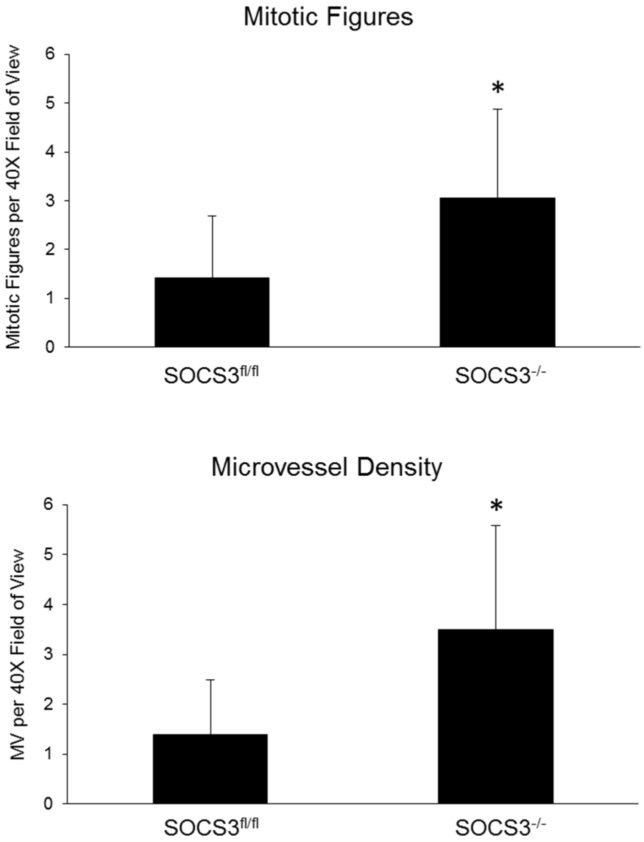

Supplementary Figure S2: Increased mitotic figures and microvessels in tumors of SOCS3$^{-/-}$ mice compared to SOCS3$^{fl/fl}$ mice at time of death from tumor burden. SOCS3$^{fl/fl}$ and SOCS3$^{-/-}$ mice were injected with GL261-Luc cells (1×10^6 cells/5 µl). At death (survival curve), brains from SOCS3$^{fl/fl}$ and SOCS3$^{-/-}$ mice were formalin fixed, paraffin embedded, sectioned and stained with Ki67 and vWf. Number of mitotic figures and vWf positive vessels were quantified (# positive per 40× field of view) in the tumors of SOCS3$^{fl/fl}$ and SOCS3 $^{-/-}$ mice. SOCS3$^{fl/fl}$ $n = 20$; SOCS3$^{-/-}$ $n = 20$. *$p < 0.05$.

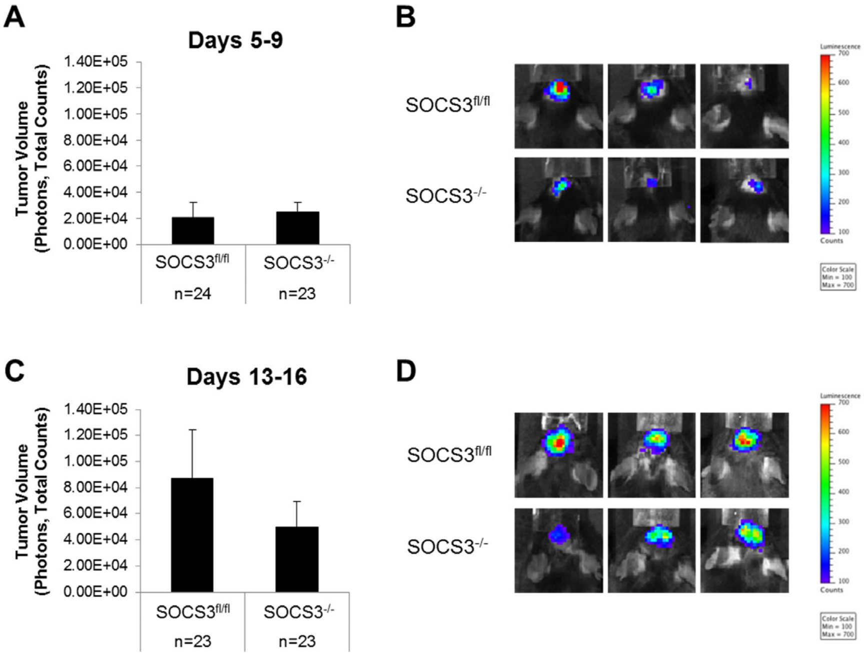

Supplementary Figure S3: Measurement of tumor volumes of SOCS3$^{fl/fl}$ and SOCS3$^{-/-}$ mice injected with GL261-Luc tumors *in vivo*. (**A**–**D**) SOCS3$^{fl/fl}$ and SOCS3$^{-/-}$ mice were injected with GL261-Luc cells (1×10^6 cells/5 µl) and bioluminescent imaging (BLI) performed. Tumor volume (total counts, photons) was quantitated and averaged for the indicated time periods (A and C) and representative images are shown for each group and time (B and D). Data are shown as mean ± S.E.M.
